# Role of Laparoscopy in Severe Gastrointestinal Bleeding Secondary to Coats Plus Syndrome

**DOI:** 10.7759/cureus.69158

**Published:** 2024-09-11

**Authors:** Miguel Serpa-Irizarry, Pedro Ruiz-Medina, Antonio Del Valle-Segarra, Jorge Zequeira-Diaz

**Affiliations:** 1 General Surgery, University of Puerto Rico, Medical Sciences Campus, San Juan, PRI; 2 General Surgery, University of Puerto Rico Medical Sciences Campus, San Juan, PRI; 3 Pediatrics, University of Puerto Rico Pediatric Hospital, San Juan, PRI

**Keywords:** cerebroretinal microangiopathy with calcifications and cyst, gastrointestinal hemorrhage, laparoscopic-assisted, intraoperative enteroscopy, coats plus syndrome

## Abstract

Coats plus syndrome (CPS) is an exceedingly rare genetic disorder associated with premature telomere shortening. The syndrome, also known as cerebroretinal microangiopathy with calcifications and cysts, has a multisystemic manifestation. It may present as brain abnormalities, seizures, osteopenia, prenatal and postnatal growth deficiency, and portal hypertension, among others. Up to 40% of affected individuals manifest recurrent gastrointestinal (GI) bleeding which can be life-threatening in some cases. Treatment for GI bleeding is not standardized and is therefore individualized based on the patient's clinical status, comorbidities, and resource availability. We herein present a case of a 20-year-old female with CPS and a two-year history of severe recurrent GI bleeding unable to be identified by conventional endoscopy. This report highlights successful laparoscopic assisted enteroscopy with enterectomy as a novel diagnostic and therapeutic modality in this population.

## Introduction

Coats Plus Syndrome (CPS) is an autosomal recessive disorder caused by mutations of the conserved telomere maintenance component 1 (*CTC1*) gene which is located on chromosome 17p13.1 and plays an essential role in telomere replication and conservation [1-4]. The result is a multisystem disorder characterized by cerebroretinal microangiopathy with calcifications and cysts. Other manifestations include retinal telangiectasias and subretinal exudate, leukodystrophy, growth deficiencies, and osteopenia [5-10]. Gastrointestinal (GI) bleeding and portal hypertension are also classic manifestations and, although frequently managed conservatively, are associated with high morbidity and mortality [5]. Since the role of surgery in the management of these patients is scarcely reported, we highlight the use of laparoscopy for the diagnosis and management of severe life-threatening gastrointestinal hemorrhage secondary to CPS.

## Case presentation

Our patient was a 20 y/o female with CTC1 mutation diagnosed with CPS, who was consulted to the pediatric surgery service due to a two year history of gastrointestinal bleeding. Previous conventional endoscopic studies had only revealed esophagitis and mild gastritis. However, the patient continued with significant anemia alternating between melena, bright red blood per rectum and positive fecal occult blood tests. While the GI bleeding progressed, she had been requiring packed red blood cell transfusions every two to three weeks during a one year period. A tagged red blood cell scan was performed and showed evidence of a blush in the left upper quadrant, unable to pinpoint the specific source of bleeding. Since multiple previous endoscopies had failed, alternative options were entertained. After informed consent, the patient was taken to the OR for a laparoscopic-assisted enteroscopy. 

Under general anesthesia, a small midline infraumbilical incision was performed and a 5 mm blunt tip trocar was introduced under direct visualization. The peritoneal cavity was insufflated and two additional 3 mm trocars were introduced under direct vision through the suprapubic area and left lower quadrant. The small bowel was inspected twice from the ileocecal valve proximally to the ligament of Treitz. No external small bowel abnormalities or Meckel’s diverticulum were identified. A portion of small bowel midway through the ligament of Treitz and ileocecal valve was selected and grasped. Then, the infraumbilical incision was extended and a small sized wound protector-retractor was placed. The previously selected portion of small bowel was exteriorized and a longitudinal enterotomy was performed at its antimesenteric border through which an endoscope was introduced. Proximal and distal enteroscopies were then performed which revealed an unremarkable distal small bowel with the exception of visible melena. At the proximal jejunum, a 5 cm area of mucosal bleeding with abundant fresh blood clots was identified. This segment was excised using a 75 mm gastrointestinal stapling device, and a side-to-side mechanical anastomosis was performed. The internal staple line was inspected for hemostasis and the enterotomy was closed transversely. No postoperative complications occurred. She remained admitted for postoperative monitoring and was discharged home on postoperative day 7 tolerating diet with stable hemoglobin levels. The pathology report showed “focal hemorrhages and vascular ectasia”. 

## Discussion

Coats Plus Syndrome is a rare genetic disorder caused by defective telomere replication [1-3]. Aside from its well-described neurologic manifestations, CPS is also characterized by recurrent gastrointestinal hemorrhage. The pathophysiology is thought to stem from vascular ectasia and other vascular abnormalities that have been identified to develop in the stomach, small intestine, and liver [1-8]. When associated with hepatic insufficiency, overt GI bleeding has also been identified to arise from esophageal varices [5]. Traditional esophagogastroduodendoscopy, colonoscopy, capsule endoscopy, double balloon enteroscopy, and mesenteric angiography are invasive modalities that have been described to locate these lesions and the source of gastrointestinal hemorrhage [5-9]. In our case, endoscopy failed to locate the hemorrhage site and further investigation was warranted. Ultimately, a tagged red blood cell scan yielded a nonspecific area of blush at the left upper quadrant. Due to diagnostic uncertainty and severe gastrointestinal hemorrhage, we opted for a more aggressive surgical approach for both diagnostic and therapeutic purposes.

Multiple modalities have been described for the treatment of gastrointestinal bleeding in patients with CPS. Most often, the methods employed are conservative in nature, and rarely has a surgical approach been described. Hoşnut and peers published a case of a CPS patient with GI bleeding secondary to multiple millimetric ulcers in the proximal small bowel that responded well to intravenous (IV) octreotide and proton pump inhibitors (PPIs) [8]. Bozkurt and colleagues described a case in which they used IV PPIs and IV octreotide for severe episodes and tranexamic acid for milder ones [7]. However, this patient eventually died due to severe GI bleeding and malnutrition. Briggs and colleagues described a case of CPS with gastric antral vascular ectasia and recurrent hematemesis that was treated with argon laser therapy and complicated by iatrogenic gastric perforation, for which a trial of estrogen and progesterone therapy was attempted [5]. They reported successful results for the first two years, nonetheless, the patient subsequently developed severe pulmonary complications and passed away. Jeraq et. al. report a similar case of CPS with GI bleeding in which multiple endoscopies failed to locate the source of bleeding, and a tagged red blood cell scan and mesenteric angiography were also unavailing [6]. Eventually, a capsule endoscopy was able to locate a source of mucosal bleeding in the proximal small bowel. Their management consisted of a combination of PPIs, octreotide, estrogen, and ortho-cyclen, but unfortunately, the patient died secondary to multiorgan failure. 

Although standardized surgical treatment has not been established, in our case, we opted for laparoscopic-assisted enteroscopy as a definitive diagnostic and eventually therapeutic method. Once the source of GI hemorrhage was able to be identified as a segment of jejunal mucosal bleeding, a small bowel enterectomy with side-to-side anastomosis was performed. The patient tolerated the procedure well and she was able to be discharged home a few days later. After which, milder episodes of GI bleeding have developed requiring occasional supportive blood transfusions. Nonetheless, these have not been sufficiently severe to be considered life-threatening or frequent enough to impair her quality of life as they had in the past.

## Conclusions

Our case aims to highlight that when the diagnosis of life-threatening GI hemorrhage secondary to CPS is still unclear, a more invasive approach to diagnosis and management should be considered. Standardized surgical management has not been established for this seldomly encountered condition with cases reported in the single digits. The infrequency and variability of symptoms make tailored recommendations difficult. With multiple reports of fatal gastrointestinal bleeding in CPS, we conclude that laparoscopic-assisted enteroscopy may be considered as a viable diagnostic and therapeutic strategy when conservative management fails.
